# Characterization of microbiomic and geochemical compositions across the photosynthetic fringe

**DOI:** 10.3389/fmicb.2023.1176606

**Published:** 2023-04-28

**Authors:** Katelyn Weeks, Elizabeth Trembath-Reichert, Grayson Boyer, Kristopher Fecteau, Alta Howells, Francesca De Martini, Gillian H. Gile, Everett L. Shock

**Affiliations:** ^1^School of Molecular Sciences, Arizona State University, Tempe, AZ, United States; ^2^School of Earth and Space Exploration, Arizona State University, Tempe, AZ, United States; ^3^School of Life Sciences, Arizona State University, Tempe, AZ, United States; ^4^NASA Postdoctoral Program Fellow at NASA Ames Research Center, Moffett Field, CA, United States; ^5^Department of Life Sciences, Mesa Community College, Mesa, AZ, United States

**Keywords:** Yellowstone National Park, hot spring, microbiome, photosynthetic fringe, geochemistry

## Abstract

Hot spring outflow channels provide geochemical gradients that are reflected in microbial community compositions. In many hot spring outflows, there is a distinct visual demarcation as the community transitions from predominantly chemotrophs to having visible pigments from phototrophs. It has been hypothesized that this transition to phototrophy, known as the photosynthetic fringe, is a result of the pH, temperature, and/or sulfide concentration gradients in the hot spring outflows. Here, we explicitly evaluated the predictive capability of geochemistry in determining the location of the photosynthetic fringe in hot spring outflows. A total of 46 samples were taken from 12 hot spring outflows in Yellowstone National Park that spanned pH values from 1.9 to 9.0 and temperatures from 28.9 to 92.2°C. Sampling locations were selected to be equidistant in geochemical space above and below the photosynthetic fringe based on linear discriminant analysis. Although pH, temperature, and total sulfide concentrations have all previously been cited as determining factors for microbial community composition, total sulfide did not correlate with microbial community composition with statistical significance in non-metric multidimensional scaling. In contrast, pH, temperature, ammonia, dissolved organic carbon, dissolved inorganic carbon, and dissolved oxygen did correlate with the microbial community composition with statistical significance. Additionally, there was observed statistical significance between beta diversity and the relative position to the photosynthetic fringe with sites above the photosynthetic fringe being significantly different from those at or below the photosynthetic fringe according to canonical correspondence analysis. However, in combination, the geochemical parameters considered in this study only accounted for 35% of the variation in microbial community composition determined by redundancy analysis. In co-occurrence network analyses, each clique correlated with either pH and/or temperature, whereas sulfide concentrations only correlated with individual nodes. These results indicate that there is a complex interplay between geochemical variables and the position of the photosynthetic fringe that cannot be fully explained by statistical correlations with the individual geochemical variables included in this study.

## 1. Introduction

In hot spring outflow channels, there is a visual transition from predominantly chemotrophic microbial communities to those with larger contributions from phototrophs. This transition is marked by the occurrence of green, orange, yellow, brown, and/or purple pigments in biofilms associated with chlorophylls, carotenoids, and/or phycobiliproteins (Cox et al., 2011). The first transition to pigmented organisms is referred to as the photosynthetic fringe (Shock and Holland, 2007). This visual transition corresponds to geochemical transitions as the hot spring water flows away from its source and begins to cool and equilibrate with the atmosphere. This process leads to more oxygenation and increased pH as CO_2_ degases, among other geochemical changes (Nordstrom et al., 2005).

Previous studies have attributed the visual appearance of the photosynthetic fringe to concurrent changes in geochemistry that are more conducive to phototrophs in hot spring outflows. Temperature, pH, and sulfide concentrations have been suggested as limiting factors of photosynthesis in hot spring outflows (Cox et al., 2011; Boyd et al., 2012; Hamilton et al., 2012). Previous work established an upper limit of photosynthesis between 73 and 75°C across environments (Kempner, 1963; Brock and Brock, 1966; Brock, 1978; Castenholz, 1969). However, the upper temperature limit of photosynthesis depends on pH (Cox et al., 2011; Boyd et al., 2012; Fecteau et al., 2022) and is reduced to ∼56°C under acidic conditions (Doemel and Brock, 1970, 1971). Though the upper temperature limit for photosynthesis was established based on observation, culture work, microbial activity, and pigment studies, the advent of sequencing methods challenges these earlier findings. Additionally, the reason for the upper temperature limit for photosynthesis is still debated. Possible limits on photosynthesis based on temperature include protein instability and the functionality of the CO_2_-assimilating mechanism (Brock and Brock, 1966; Meeks and Castenholz, 1978). It is also uncertain why the temperature limit is lower in acidic conditions, but it is most likely due to the dominant phototrophs transitioning from bacteria to comparatively less thermotolerant eukaryotes at lower pH (Boyd et al., 2012; Fecteau et al., 2022). Sulfide concentration may also be a limiting factor for photosynthesis due to sulfide’s ability to bind to metalloproteins and block electron flow to photosystem II (Oren et al., 1979; Miller and Bebout, 2004).

In the phototrophic communities of hot spring outflows in Yellowstone National Park (YNP), the composition of phototrophs changes with pH. In the phototrophic mats below the photosynthetic fringes of basic springs (pH > 7), the microbial communities consist predominantly of bacterial phototrophs, including Cyanobacteria and filamentous anoxygenic phototrophs (Inskeep et al., 2013; Bennett et al., 2022). The predominance of bacterial phototrophs in basic hot spring outflows has been supported by 16S rRNA gene sequencing, metagenomic sequencing, and *in situ* studies of bicarbonate and nitrogen fixation (Ward et al., 1990; Steunou et al., 2008; Klatt et al., 2011; Thiel et al., 2016). Below the photosynthetic fringe of acidic outflows (pH < 4), the phototrophs are typically eukaryotic and include acidophilic algae such as *Cyanidioschyzon* (Toplin et al., 2008; Skorupa et al., 2013). Both eukaryotic and bacterial phototrophs have been identified in the phototrophic mats of acidic to circumneutral hot springs (pH 4–7), combining the likes of Cyanobacteria and *Cyanidioschyzon* (Fecteau et al., 2022). Thus, in hot spring environments there exists a trend in microbial community composition from prokaryotic to eukaryotic phototrophs as pH decreases (Brock, 1973; Bennett et al., 2022; Fecteau et al., 2022).

The predominantly chemotrophic communities above the photosynthetic fringe of hot spring outflows also vary with pH (Swingley et al., 2012; Inskeep et al., 2013; Colman et al., 2016; Lindsay et al., 2018). In general, according to quantitative PCR amplification of 16S rRNA genes, Archaea dominate in the chemotrophic communities of acidic hot springs, whereas Bacteria are dominant in the chemotrophic communities of basic hot springs (Colman et al., 2018). In acidic hot springs (pH < 4), predominant archaeal constituents are Sulfolobales, Desulfurcoccales, and Thermoproteales (Inskeep et al., 2013; Colman et al., 2018). The predominant bacteria in acidic hot springs include Aquificales, Thermales, Firmicutes, and Proteobacteria (Inskeep et al., 2013; Colman et al., 2016). However, it should be noted that Aquificales and Proteobacteria are predominant bacterial constituents in hot spring outflows regardless of pH. These previous studies have provided characterizations of chemotrophic community compositions and implicate the importance of pH on the composition of the community.

The distinct compositions of microbial communities along hot spring outflows have been linked to differences in the concentrations of dissolved inorganic carbon (DIC) and dissolved organic carbon (DOC). Havig et al. (2011) investigated the presence versus absence of various carbon fixation pathways and connected the findings back to changes in carbon isotope fractionation (Δ^13^C) data as well as DIC and DOC concentrations. In the outflow of a basic spring, “Bison Pool,” also discussed in this study as hot spring “BP,” Δ^13^C measurements of the biofilm became more negative down the outflow, indicating a possible shift in carbon fixation strategies (Havig et al., 2011). Additionally, DIC was observed to decrease down the outflow while DOC increases. Generally, DIC concentrations are dependent on CO_2_ input from the hydrothermal source and decrease down hot spring outflows as CO_2_ degasses and is microbially fixed. In contrast, DOC generally increases down the outflow, and, in the case of “Bison Pool,” this is attributed to meteoric water input from the surrounding meadow (Swingley et al., 2012). In the “Bison Pool” outflow, the increase in DOC was connected to a transition in the microbial community composition from chemoautotrophs at the highest temperatures to heterotrophs and phototrophs further downstream, implicating the importance of DIC/DOC in the composition of the microbial community (Swingley et al., 2012).

Nitrogen availability in YNP hot spring outflows also contributes to the microbial community composition. According to isotopic observations by Havig et al. (2011), measurable N-fixation only occurs at and below the photosynthetic fringe of the “Bison Pool” outflow. This limitation on N-fixation was reflected in the distribution of nitrogen fixation (*nif*) genes only at and below the photosynthetic fringe. In contrast, the expression of *nif* genes was observed above the photosynthetic fringe at “Mound Spring,” also discussed in this study as hot spring “MN” (Loiacono et al., 2012). *nif* genes have also been identified in acidic to circumneutral springs, suggesting that nitrogen fixation is not limited by pH in hot spring ecosystems (Hamilton et al., 2011a). This was supported further by enrichment of diazotrophs from acidic YNP hot springs that were shown to fix nitrogen *in situ* via acetylene reduction assays (Hamilton et al., 2011b). In contrast, *amoA*, a gene associated with ammonia oxidation, is predominantly found in circumneutral to basic hot springs, and ammonia-oxidizers have only been enriched from circumneutral to basic YNP hot springs (De la Torre et al., 2008; Hatzenpichler et al., 2008; Hamilton et al., 2011b; Boyd et al., 2013). It is hypothesized that ammonia-oxidizers outcompete diazotrophs in circumneutral to basic hot springs, consuming the bioavailable nitrogen, thereby producing a downstream niche for diazotrophs (Hamilton et al., 2014). Genetic and geochemical analyses both implicate nitrogen as a determining factor for microbial community composition in YNP hot spring outflows.

In this study, 12 hot spring outflows in YNP were selected for sampling above, at, and below the photosynthetic fringe spanning temperatures from 28.9 to 92.2°C, pH from 1.9 to 9.0, and sulfide concentrations from below the detection limit (<0.15 μmolal) to 52.6 μmolal (Supplementary Table 1). Due to this sampling scheme, each of the 12 hot spring outflows differ in both geochemical and microbiomic diversity and complexity, in addition to differing in history and geographic location. Site selection used linear discriminant analysis (LDA) of multiple geochemical parameters to estimate equidistant geochemical space above and below the visual photosynthetic fringe, for a total of 46 samples. Communities in sample sites above the photosynthetic fringe were expected to consist predominantly of chemotrophs, while those below the photosynthetic fringe were expected to contain a larger contribution of phototrophs. Samples at the photosynthetic fringe provided insight into the transition between the predominantly chemotrophic to the phototroph-containing microbial communities. From each sample site, geochemical measurements were taken including temperature, pH, conductivity, total sulfide, total dissolved silica, ferrous iron, dissolved oxygen gas (DO), DOC, DIC, and major cation and anion concentrations (Supplementary Tables 1, 2). In addition, 16S rRNA gene sequencing was performed using a sediment slurry from each sample location to determine the microbial community composition along the hot spring outflows (Supplementary Table 3). In combination, the geochemical measurements and sequencing data provide insights into how both the microbial community and the geochemistry change and interact down hot spring outflows and across the photosynthetic fringe. However, we find collapsing the complexity of these hot spring outflows into a list of geochemical variables was insufficient to determine the exact position of the photosynthetic fringe in geochemical space.

## 2. Materials and methods

### 2.1. Sample site selection via LDA

Sampling locations (Supplementary Table 1) were determined by linear discriminant analysis (LDA), in which at least three samples were collected to represent locations *below* (chemosynthetic and photosynthetic), *at* (fringe), and *above* (chemosynthetic) the photosynthetic fringe in hot spring outflow channels, though additional samples were taken at several sites to provide additional biogeochemical context. The LDA model (Equation 1) was trained to separate samples into photosynthetic and non-photosynthetic classes based on 20 variables measured across 56 samples collected from twenty-nine geochemically diverse hot springs in previous years. The 20 variables selected to construct the LDA model were chosen based on perceived biological relevance, specifically, temperature, pH, conductivity, DIC, DOC, DO, Fe(II), sulfide, phosphate, total ammonia, and total dissolved Mg, Co, Ni, Cu, Zn, As, Mo, Cd, W, and Pb. Fe(II) was used in place of total Fe because Fe(II) could be measured in the field spectrophotometrically. Additionally, representative variables were chosen per element, for example, total ammonia is representative for nitrogen species. Samples above the photosynthetic fringe tend to have greater negative LDA scores, while samples below tend to have greater positive scores. Photosynthetic fringe positions were determined visually when possible and by LDA when not discernable by eye. When testing the LDA model on 381 previously collected samples, where photosynthesis had been identified by the visual presence of photosynthetic pigments, an LDA score of 0.13 predicted photosynthetic and non-photosynthetic samples with the fewest number of false positives and negatives. In outflow channels where the fringe was not apparent, the location of the fringe was estimated by choosing a location where the LDA score was close to or equal to 0.13 based on temperature, pH, and conductivity measured in the field combined with historical data for the remaining 17 variables in the LDA model. In the outflow of CF and MO, the photosynthetic fringe was apparent but occurred at different temperatures throughout the hot spring outflow, hence multiple samples were taken throughout the outflows to account for these variations. Sampling locations were chosen such that they were chemically equidistant from the fringe as estimated by the LDA model. In other words, sampling was carried out such that the difference in LDA scores between the *at* and *below* samples was equal to the difference between *above* and *at* scores. The LDA model was trained using the lda function in the MASS package in R (RRID:SCR_019125) (Venables and Ripley, 2002; R Core Team, 2013).

Equation 1.


Line 1:LDAscore=1.028E-1pH+ 6.769E-5conductivity

(μS)- 6.12E-2temperature(°C)

Line⁢ 2:+2.037⁢E-4⁢DIC⁢(mg⁢C/L)+7.199⁢E-3⁢DOC

(mg⁢C/L)+1.746⁢E-1⁢DO⁢(ppm)

L⁢i⁢n⁢e⁢ 3:- 2.610⁢E-2⁢F⁢e⁢(I⁢I)⁢(p⁢p⁢m)- 9.36⁢E-5⁢Σ⁢H⁢S-⁢(p⁢p⁢b)

$$-2.242\,E^{-2}\,\Sigma \,P\,O_{4}^{3-}\,\left(p\,p\,m\right)$$

$$Line\;4:\;-\;1.615E\;-\;3\;\Sigma NH_{4}^{+}\;(ppm)\;-\text{​}2.57E\;-\;5\;\Sigma Mg\;(ppb)$$

- 2.01⁢E-2⁢Σ⁢C⁢o⁢(p⁢p⁢b)

L⁢i⁢n⁢e⁢ 5:+ 2.39⁢E-2⁢Σ⁢N⁢i⁢(p⁢p⁢b)+ 2.89⁢E-3⁢Σ⁢C⁢u⁢(p⁢p⁢b)

L⁢i⁢n⁢e⁢ 6:- 2.77⁢E-3⁢Σ⁢Z⁢n⁢(p⁢p⁢b)- 2.35⁢E-4⁢Σ⁢A⁢s⁢(p⁢p⁢b)

- 6.35⁢E-3⁢Σ⁢M⁢o⁢(p⁢p⁢b)

L⁢i⁢n⁢e⁢ 7:- 7.15⁢E-1⁢Σ⁢C⁢d⁢(p⁢p⁢b)+ 2.44⁢E-3⁢Σ⁢W⁢(p⁢p⁢b)

- 2.35⁢E-2⁢Σ⁢P⁢b⁢(p⁢p⁢b)

Sigma symbols indicate that the variable includes measured values of total solute concentrations and includes all protonation states and complexes.

### 2.2. Geochemical sampling and analyses

Temperature, pH, and conductivity were measured in the field as previously described (Boyer et al., 2020). Temperature and conductivity were measured using a YSI-30 portable meter (YSI, Yellow Springs, OH, USA). Measurements of pH were obtained using a WTW 3110 meter and SenTix 41 temperature-compensated probes (Xylem Analytics, Weilheim, Germany) calibrated daily at ambient temperature using buffered pH solutions. Dissolved oxygen was measured optically using a PreSens Fibox 4 meter and a DP-PSt3-L2.5-St10-YOP-HT sensor calibrated to 100°C (PreSens, Regensburg, Germany) as previously described (St Clair et al., 2019). Total dissolved sulfide was determined via the methylene blue method using Hach reagents and a DR1900 spectrophotometer on unfiltered water samples and analyzed immediately after collection.

Filtered (0.2 micron; Supor, Pall Corporation, Port Washington, NY, USA) water samples for laboratory analyses were collected and stored according to previously described procedures (Fecteau et al., 2022). Samples for anions were collected in 30 ml high-density polyethylene (HDPE) bottles that had been soaked and rinsed with deionized water multiple times; separate 30 ml samples for cations were collected in bottles that had been spiked with 6 M methanesulfonic acid resulting in a final concentration of ∼20 mM. These samples were frozen at –20°C as soon as possible after collection and maintained at that temperature until analysis. DIC samples were collected in acid-washed 40 ml amber class vials and sealed with black butyl rubber septa without any headspace. DOC samples were collected in combusted (450°C, 24 h) 40 ml amber glass vials spiked with 0.1 ml of 85% phosphoric acid (Thermo Scientific, Waltham, MA, USA) and sealed with Teflon-lined septa without any headspace.

Anions (F^–^, Cl^–^, SO_4_^–2^, Br^–^, NO_3_^–^) and cations (Li^+^, Na^+^, K^+^, Mg^+2^, Ca^+2^, NH_4_^+^) were determined on separate Dionex DX-600 4 mm ion chromatography systems using suppressed-conductivity detection as described elsewhere (Iacovino et al., 2020). Samples were injected via AS-40 autosamplers from 5 ml vials (2 injections per vial) onto 100 μl or 75 μl sample loops for anions or cations, respectively. Anions were separated using AG-/AS-18 columns and a hydroxide concentration gradient that was initially held isocratically at 5 mM for 10 min, followed by a non-linear (Chromeleon curve 8) (RRID:SCR_016874) gradient applied over 32 min to 55 mM hydroxide, after which the concentration was kept constant at 55 mM for 7 min, reduced back to 5 mM hydroxide over 1 min, and then the column was re-equilibrated at 5 mM hydroxide for 10 min before the next sample injection. The flow rate was held constant at 1 ml/min. Cations were separated isocratically using 19 mM methanesulfonic acid on CG-/CS-16 columns at 0.5 ml/min over 58 min. Suppressors were operated in external water mode and suppressor currents were 137 and 50 mA for anions and cations, respectively. Calibration curves were constructed from a series of dilutions of mixed-ion standards (Environmental Express, Charleston, SC, USA) and accuracy was verified daily by analysis of an independent mixed-ion standard (Thermo Scientific).

Analyses of DIC and DOC were performed with a OI Wet Oxidation TOC analyzer coupled to a Thermo Delta Plus Advantage mass spectrometer as previously described (Havig et al., 2011). Briefly, CO_2_ was generated via addition of phosphoric acid (DIC) or sodium persulfate (DOC) and the ion chromatogram for the molecular ion (44 m/z) was used for quantification relative to calibration curves prepared with sodium bicarbonate (DIC) or glycine (DOC) standards. Three sample loops with volumes of 1 ml (calibration range 10–200 mg C L^–1^), 5 ml (calibration range 2–50 mg C L^–1^), and 25 ml (calibration range 0.25–8 mg C L^–1^) were employed to capture the range of carbon concentrations across the sample set.

### 2.3. Biological sampling, extractions, and sequencing

Hot spring outflow sediment samples were collected and preserved for biological analyses (16S rRNA gene amplicon sequencing and subsequent microbial community diversity analyses). Samples were collected using a flame-sterilized spatula into a 1.8 ml cryovial. Once samples were collected, they were transferred into a container of dry ice and frozen until they could be stored at −80°C at ASU.

For DNA extraction, biological samples were homogenized, and DNA was extracted using a ZymoBIOMICS DNA Miniprep Kit (Catalog # D4300), binding capacity 25 μg, as previously described (Howells, 2020). A NanoDrop was used to spectrophotometrically analyze the purity of the DNA and a Qubit fluorometric assay kit from Invitrogen (Catalog # Q32850) was used to determine the concentration of purified DNA. The DNA was then sequenced for both bacterial and archaeal 16S rRNA gene amplicons at Arizona State University’s Biodesign Institute using Illumina MiSeq v2 2 × 300 chemistry (RRID:SCR_020134) with the Earth Microbiome Project primers 505F and 806R (Thompson et al., 2017). The 16S rRNA gene amplicon library was prepared following Earth Microbiome Project protocol,^1^ (Caporaso et al., 2012). All raw sequences were uploaded to the NCBI Sequence Read Archive (SRA) (RRID:SCR_004891) under BioProject ID PRJNA938133.

### 2.4. Bioinformatic analyses

FASTQC (v. 0.11.9) (RRID:SCR_014583) was used to quality filter the 16S rRNA amplicon sequences (Andrews, 2010). The resulting high quality fasta files were then processed using the QIIME2 (v. 2020.2) (RRID:SCR_021258) pipeline to produce amplicon sequence variants (ASVs) and were denoised using the DADA2 plug-in (Bolyen et al., 2019). The SILVA database (RRID:SCR_006423) was used for the taxonomic classification of ASVs (Quast et al., 2012). The produced ASV table was normalized by putting the sequence counts into relative abundances and multiplying them by the mean library size (Supplementary Table 3; Fullerton et al., 2021). Non-metric multidimensional scaling (NMDS) analyses were performed using VEGAN R software (v. 2.5-7) (RRID:SCR_011950) and Bray-Curtis Dissimilarity values (Oksanen et al., 2022). The envfit function from the VEGAN package was used to add geochemical vectors to the NMDS and to calculate the respective *p*-values for each geochemical vector. The percent contribution for each geochemical vector was determined using the redundancy analysis (RDA) function in the VEGAN package. To determine the significance of separating sites as above, at, or below the photosynthetic fringe, analysis was performed using the canonical correlation analysis (CCA) function from the VEGAN package and an ANOVA test was performed on each axis using the R stats package. For the co-occurrence network analysis, ASVs were filtered to only include ASVs that had more than 20 reads across all samples and occurrences across more than 3 sample sites. The remaining ASVs were used to construct a co-occurrence network using R’s igraph package (v. 1.2.11) (RRID:SCR_021238), in which each node is an ASV, and each edge represents a Spearman’s correlation coefficient greater than 0.7 between the two nodes (Csardi and Nepusz, 2006; Fullerton et al., 2021). Cliques were determined by using the Louvain membership algorithm (Csardi and Nepusz, 2006). The nodes were then plotted and colored in Gephi (v. 0.9.4) (RRID:SCR_004293) based on their Spearman correlation with the selected geochemical variables (Bastian et al., 2009).

## 3. Results

### 3.1. Sampling locations and geochemical data

A total of twelve hot springs from eight separate locations within YNP were sampled to investigate the connection between the microbiomic and geochemical transitions that occur down hot spring outflows (Supplementary Table 1 and Supplementary Figure 1).

**FIGURE 1:**
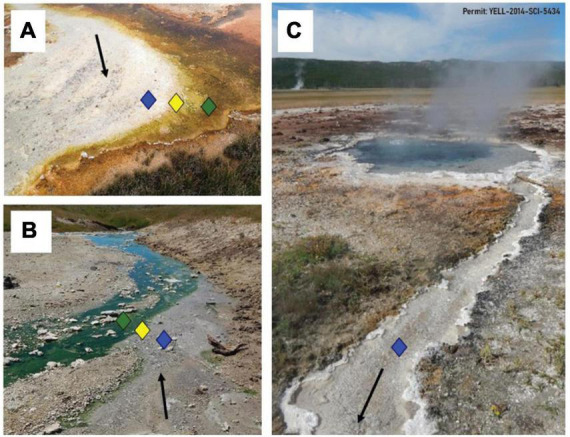
Hot spring outflows with depictions of the photosynthetic fringe. Locations above (blue), at (yellow), and below (green) the photosynthetic fringe are indicated by respective diamonds. Black arrows indicate a general flow of water away from the source. **(A)** The visual representation of the photosynthetic fringe in the outflow of a basic spring, BP, where the white sinter in the outflow meets orange phototrophic mats along the edge of the outflow. **(B)** The visual representation of the photosynthetic fringe in the outflow of an acidic spring, CF, where the clear outflow meets green phototrophic mats. **(C)** An example of the start of a hot spring outflow channel from a basic hot spring, BP, before reaching its photosynthetic fringe.

To select sample sites, an LDA equation was developed (see section 2. Materials and methods) to determine the locations above and below the photosynthetic fringe such that sampling would be equidistant from the fringe in multivariate geochemical space. The photosynthetic fringe can often be visually confirmed as seen in example images of a sampled basic (Figure 1A) and an acidic (Figure 1B) hot spring outflow channel. An example of a sampled hot spring outflow before reaching the photosynthetic fringe is shown in Figure 1C. In 3 of the 12 hot spring outflows, the photosynthetic fringe could not be visually identified, so temperature, pH, and conductivity measurements were taken along the hot spring outflow and used in the LDA model to predict the location of the photosynthetic fringe.

**FIGURE 2:**
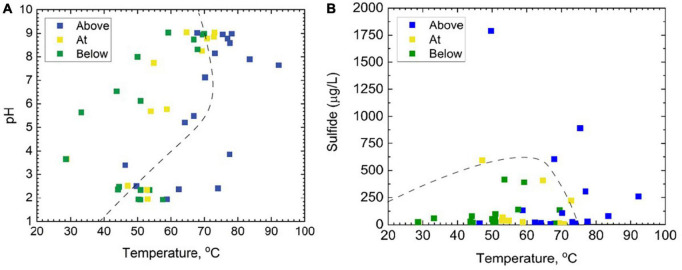
A total of 46 samples taken from the outflows of 12 separate hot springs above, at, and below the photosynthetic fringe, as determined by linear discriminant analysis, displayed as functions of **(A)** pH and temperature, and **(B)** total dissolved sulfide and temperature. In both panels **(A,B)**, the dashed line represents the photosynthetic limits defined by Cox et al. (2011).

In total, 46 samples were taken with the intention of spanning the temperature, pH, and sulfide ranges provided by YNP hot springs (Figure 2 and Supplementary Table 1). Of the 12 hot spring outflows sampled, four were acidic (pH < 4) (CF, MO, GL, and CH), three were acidic to circumneutral (pH 4–7) (MU, FI, and EM), and five were considered basic (pH 7–9) (RN, OB, BP, PB, and MN). Additionally, Shannon diversity values were computed to assess the alpha diversity of the microbial community and range from 2.63 to 5.93 (Supplementary Table 1). This study includes additional geochemical measurements of total ammonia, nitrate, DIC, DOC, and DO concentrations (Supplementary Table 1). Of the major ions measured, total ammonia and nitrate were the two most significant ions, contributing 3.78 and 2.55%, respectively, to overall microbial community composition (Supplementary Table 4) determined via the VEGAN package in R using RDA. Total ammonia concentrations span a range of 0.63–1171 μmolal, whereas nitrate concentrations are 0.07–1.01 μmolal. DOC and DIC concentrations span a range of 0.20–1.89 mg C/L, and 0.04–66.98 mg C/L, respectively. All Pearson correlations between the geochemical measurements from each sample are included in Supplementary Figure 3. According to the Pearson correlation values, there are nine strongly correlated (> 0.50) geochemical parameters. There are strong positive correlations between temperature and pH (0.58), temperature and DIC (0.71), pH and DIC (0.79), and ammonia and DO (0.53). Strong negative correlations include temperature with total ammonia (−0.61), temperature with DO (−0.78), pH with ammonia (−0.57), pH with DOC (−0.77), ammonia with DIC (−0.53), and DIC with DOC (−0.68). There are no strong positive or negative correlations between sulfide or nitrate concentrations and any of the other geochemical parameters.

### 3.2. Microbial community composition

**FIGURE 3:**
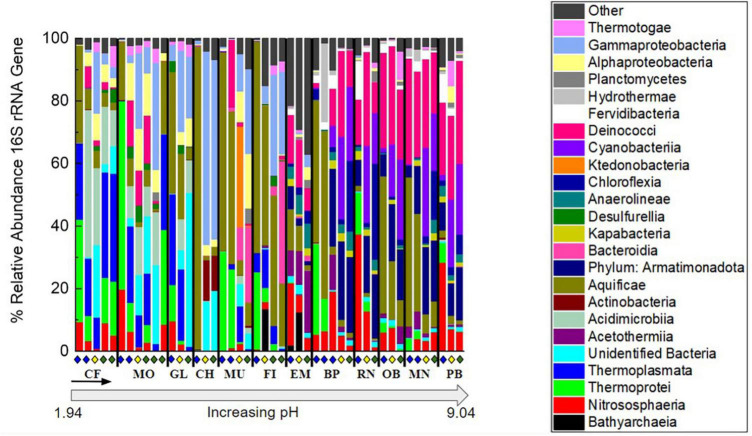
Percent relative abundance of the 16S rRNA gene sequencing results to the class level except for Armatimonadota, which is at the phylum level, and unidentified bacteria, which were binned together at the domain level. To focus on abundant features and overarching patterns, classes not occurring at >20% relative abundance when summed over all samples were binned into the “Other” category. Organization of hot spring sites, separated by black bars, follows the order of increasing pH shown in Supplementary Table 1. Within each site, samples are organized down the outflow with above (blue diamond), at (yellow diamond), and below (green diamond) the photosynthetic fringe indicated as in Figure 1.

The relative abundance of taxonomic classes present in each of the 46 samples was determined by 16S rRNA gene sequencing to investigate the microbial community composition and diversity along each outflow and among the hot springs sampled (Figure 3). In each of the hot springs sampled, ASVs associated with the taxonomic classes Thermotogae, Planctomycetia, Deinococci, Aquificae, Thermoprotei, and Nitrososphaeria were present. Aquificae and Deinococci were present in high relative abundances across all hot springs, up to 96.8 and 33.0%, respectively. In the four most basic springs sampled (RN, OB, MN, and PB), Deinococci reached relative abundances greater than 30%. Additionally, Nitrososphaeria occurred across all hot springs sampled with the highest relative abundances occurring in the basic sites, RN and OB, and above the photosynthetic fringe with relative abundances of 37.4 and 28.4%, respectively. Both Thermotogae and Planctomycetia occurred at all hot spring sites but had consistently low relative abundances, ranging from around 0.1–9.0 and 0.1–4.9%, respectively, when present. Additionally, there were unidentified bacteria present in each of the hot springs sampled with the highest relative abundances in acidic hot springs. As an example, below the photosynthetic fringe of GL, where the pH is 2.5, unidentified bacteria made up 21.2% of the community.

Amplicon sequence variants associated with photoautotrophs, including those in the phyla Cyanobacteria and Chloroflexi, such as *Leptococcus* and *Chloroflexus*, respectively, occurred with higher relative abundances in the circumneutral to basic hot spring outflows. Both Cyanobacteria and Chloroflexi occurred in nearly all hot spring outflows sampled, except, neither Cyanobacteria nor Chloroflexi occurred in the outflow of GL (49.7 °C, pH 2.5) or MO (74.0°C, pH 2.4), nor did Chloroflexi occur in the outflow of FI (64.1 °C, pH 5.2). Furthermore, neither Cyanobacteria nor Chloroflexi surpassed a relative abundance of 0.1% in the outflow of any of the acidic hot springs sampled. Chloroflexi reached relative abundances above 1% only at pH values greater than 7, while Cyanobacteria reached a relative abundance above 1% only at pH values greater than 8. In basic conditions, Chloroflexi and Cyanobacteria made up to 30.7 and 25.6% of the microbial community, respectively, at their highest relative abundances. Chloroflexi occurred above, at, and below the visually determined photosynthetic fringe. However, Chloroflexi reached higher relative abundances at (0.9–2.4%) and below (0.0–22.6%) the photosynthetic fringe compared to above (0.0–3.4%). Cyanobacteria were also identified above the visually detected photosynthetic fringe but did not surpass a relative abundance of 1.8% except at outflow sample BP0.5, where the relative abundance was 6.2%. In contrast, at and below the photosynthetic fringe, the relative abundances for Cyanobacteria made up to 25.6% of the microbial community. ASVs associated with putative photoheterotrophs were also present, including those in the taxonomic classes of Alphaproteobacteria and Acidobacteriia, such as *Acidiphilium, Acidisphaera*, and *Chloracidobacterium*, which, when combined, only surpass a relative abundance of 1% in a single sample, MU3, where they make up 4.0% of the community.

Samples at or below the photosynthetic fringe indicate an apparent cut-off for photosynthesis at ∼73°C for basic to circum neutral pH samples (Figure 2A). The temperature cut-off for photosynthesis is lower, ∼56°C, for the outflow of acidic hot springs (Figure 2A), consistent with previous studies (Cox et al., 2011; Boyd et al., 2012; Fecteau et al., 2022). However, it should be noted that there are samples categorized as being “above” the photosynthetic fringe that are within these temperature limits, indicating additional factors may be restricting the growth of photosynthetic organisms in individual locations. Common bacterial phototrophs, such as Cyanobacteria and Chloroflexi, are abundant in basic hot spring outflows at and below the photosynthetic fringe, but also occur in small relative abundances above the visually determined photosynthetic fringe (Supplementary Figure 2). None of the 46 sample sites that were below the photosynthetic fringe had total sulfide concentrations that exceeded 500 μg/L; however, there was one sample, GL2, assessed to be “at” the photosynthetic fringe that exceeded 500 μg/L, the suggested sulfide limit defined by Cox et al. (2011) (Figure 2B).

In addition to ASVs associated with photosynthetic taxa occurring in the circumneutral to basic hot spring outflows, ASVs associated with non-phototrophs in the taxonomic classes of Acetothermia, Kapabacteria, Fervidibacteria, Hydrothermae, Anaerolineae, and the phylum Armatimonadota were also present. Although Cyanobacteria and Chloroflexia were abundant within the outflow of basic hot springs, ASVs associated with the non-photosynthetic phylum Armatimonadota were also prevalent in these samples, occurring with an average relative abundance of 13.5% when present. Armatimonadota were present in every sample above a pH of 7, except for sample site 0.5 from BP. As for the ASVs associated with the taxonomic classes Acetothermia, Kapabacteria, Fervidibacteria, Hydrothermae, and Anaerolineae, they reached relative abundances above 1% only above a pH of 7 but none were as consistently predominant as ASVs associated with the phylum Armatimonadota.

The ASVs abundant in the lower pH sites (pH < 7) include ASVs from the following taxonomic classes: Gammaproteobacteria, Alphaproteobacteria, Desulfurellia, Actinobacteria, Acidimicrobiia, and Thermoplasmata. Gammaproteobacteria, Alphaproteobacteria, and Actinobacteria were present in at least one sample from each hot spring outflow but reached relative abundances above 1% only at acidic sites. Acidimicrobiia were present in all hot springs sampled except for in the outflow of MN (75.4°C, pH 9.0) and only reached a relative abundance over 10% at pH values below 3. Gammaproteobacteria had a high relative abundance of 61.8% at the photosynthetic fringe of hot spring CF where the temperature was 28.8°C and the pH was 3.7. Thermoplasmata and Desulfurellia were restricted to circumneutral and acidic sites, only occurring below a pH of 8.0 and 6.5, respectively, with Desulfurellia only making up to 5.3% of any microbial community composition, while Thermoplasmata reached up to 34.5% of the relative abundance of the microbial community composition below the photosynthetic fringe of CF.

Additionally, there were ASVs associated with three taxonomic classes that were restricted to circumneutral conditions, including Ktedonobacteria, Bacteroidia, and Bathyarchaea. Ktedonobacteria only occurred with a relative abundance higher than 1% at the photosynthetic fringe of MU, where they made up 32.2% of the community, which is at a pH of 5.7. Bacteroidia occurred across pH, but only made up more than 1% of the community in the outflows of MU, FI, and EM, which range in pH from 3.9 to 8.0. Below the photosynthetic fringe of FI, Bacteroidia made up 39.0% of the microbial community. Bathyarchaea were present in small relative abundances (<0.1%) in the outflows of RN and CF but occurred with relative abundances of 13.6 and 12.6% at the photosynthetic fringes of FI and EM, respectively. The photosynthetic fringe of FI occurred at a pH of 5.8 while the photosynthetic fringe of EM occurred at a pH of 7.8.

Overall, there were trends in microbial community composition across pH as well as down the outflows of the 12 sampled hot springs. In the lower pH sites, ASVs associated with taxonomic classes such as Thermoplasmata and Gammaproteobacteria dominated. In basic sites, ASVs associated with the phylum Armatimonadota dominated, as well as ASVs associated with the potentially photosynthetic phyla, Cyanobacteria and Chloroflexi. The relative abundance of potential bacterial photosynthetic taxa increased in relative abundance with increasing pH and increased down hot spring outflows. In contrast, the relative abundance of the taxonomic class Hydrothermae decreased down hot spring outflows. At all hot springs and outflows, ASVs for the taxonomic classes Aquificae and Deinococci were present and made up a considerable portion (19.0 and 10.8% when averaged across all samples, respectively) of the microbial community composition.

### 3.3. Geochemical influence on community composition

**FIGURE 4:**
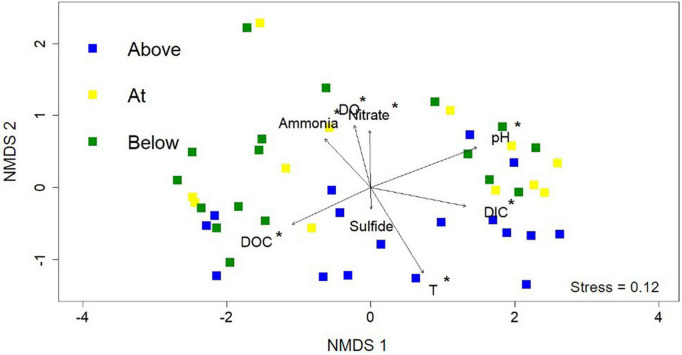
Non-metric multidimensional scaling (NMDS) analysis using the 16S rRNA gene sequencing data from each of the 46 samples. Each point represents the normalized microbial community composition determined in a hot spring sample while the distance between points represents the dissimilarity. Sample point colors (blue, yellow, and green) refer to the position along the photosynthetic fringe (above, at, below, respectively). Geochemical data are added as vectors; vectors that correlate with an ordination axis with a *p*-value < 0.05 are indicated by an asterisk.

Non-metric multidimensional scaling (NMDS) analysis was used to interrogate the influence of geochemistry on microbial community composition across the photosynthetic fringe (Figure 4). The 16S rRNA gene sequencing data was used to determine the microbial community composition (Supplementary Table 5). Geochemical vectors for each of the geochemical measurements were initially added to indicate potential causes for differences in microbiome composition in the 46 sampled locations; however, only a subset of eight geochemical vectors were included due to either their hypothesized importance (sulfide), or their contribution to the variation of the microbial community composition determined by RDA (pH, Temperature, DIC, DOC, nitrate, total ammonia, and DO) (Supplementary Table 4). Of the eight included geochemical vectors, all correlated with changes in microbial community composition with statistical significance (*p* > 0.05) except for sulfide. Along NMDS2, the distribution of sites can be differentiated based on position relative to the photosynthetic fringe. However, only sites labeled as being above the photosynthetic fringe can be differentiated from the sample sites at or below the photosynthetic fringe with statistical significance according to CCA analysis (Supplementary Figure 5). The geochemical vectors that correlate with changes in microbial community composition along NMDS2 with statistical significance are ammonia, DO, temperature, and nitrate, all of which increase down the hot spring outflows. There is more variation in microbial community composition across NMDS1 which correlates with changes in pH, DIC, and DOC with statistical significance. This indicates that there is a complex interplay between geochemical parameters and microbial community composition.

While NMDS analyses provide insight into overall microbial community composition in relation to geochemical parameters, co-occurrence network analyses provide insight at the level of individual ASVs and their correlation(s) with geochemical parameters (Fullerton et al., 2021). In a co-occurrence network, each node is an ASV. All nodes were grouped into 18 unique cliques (statistically significant groups of ASVs) using the Louvain algorithm and consisted of at least two nodes (Supplementary Table 6; Fullerton et al., 2021). Clique analysis provides a mechanism for observing microbial patterns within guilds and individuals rather than as an entire assemblage (e.g., NMDS).

**FIGURE 5:**
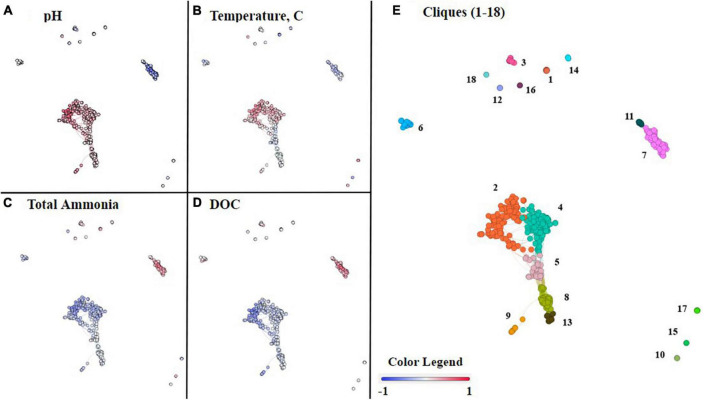
Co-occurrence network analysis of commonly occurring ASVs within the 46 hot spring samples. Each node represents an ASV, and nodes were organized into 18 cliques using Louvain’s membership algorithm. The connections between nodes, also known as edges, represent a >0.7 Spearman’s correlation coefficient. In panels **(A–D)**, each node is colored on a gradient of blue to red based on its Spearman’s correlation, from negative one to positive one, respectively, with the following geochemical parameters, **(A)** pH, **(B)** temperature, **(C)** total ammonia, and **(D)** DOC. Each of the cliques is differentiated by color and number in panel **(E)**.

The co-occurrence network consisted of ten cliques with no interconnections (modular cliques), a central cluster consisting of six interconnected cliques, and a separate cluster of two cliques. This topology suggests there are groups of ASVs that uniquely co-occur (modular cliques) and groups of ASVs that co-occur predominantly with each other (main cluster cliques). Some members of the main cluster cliques co-occur with ASVs outside the clique as well. The statistical association of the cliques with geochemical parameters of interest was conducted by Spearman correlation. From these combined analyses we can observe how cliques and individual ASVs co-varied with pH, temperature, total ammonia, DIC, DOC, nitrate, and sulfide concentrations (Figure 5; Supplementary Figure 6).

Within the main cluster of six cliques (2, 4, 5, 8, 9, and 13) all ASVs correlated positively with an increase in pH (Figure 5A). The remaining cliques, outside the central cluster, are composed of ASVs that mostly occur in acidic or circumneutral samples, and, therefore, either correlate negatively or have no significant correlation with increasing pH. However, cliques 17 and 18 are an exception, and like the main cluster cliques, are correlated with an increase in pH.

The main cluster cliques consist of the phototrophs *Leptococcus*, *Roseiflexus*, and *Chloroflexus*, as well as non-phototrophs from the taxonomic classes Aquificae, Anaerolineae, Deinococci, Acetothermiia, and the phylum Armatimonadota among others listed in Supplementary Table 6. Due to the prevalence of Aquificae and Deinococci throughout the dataset, it is important to note that the genera present in the main cluster cliques are *Thermocrinis* and *Thermus*, respectively. Although all 6 cliques within the main cluster are correlated positively with an increase in pH, only cliques 2, 4, and 9, which include ASVs associated with the taxa *Thermocrinis*, Deinococci, *Leptococcus*, Acetothermia, and Armatimonadota, are positively correlated with temperature. Clique 5 is correlated slightly negatively with temperature and consists of the taxonomic classes Anaerolineae, Acetothermia, and the phylum Armatimonadota. Additionally, cliques 2, 4, and 9 are all correlated negatively with total ammonia, whereas the nodes in clique 5 are mixed between positive and negative correlations with total ammonia. The remaining cliques in the cluster (8 and 13) are not correlated with total ammonia. In contrast, all 6 cliques within the cluster are correlated negatively with DOC, although cliques 2 and 9 have a stronger negative correlation with DOC than cliques 4, 5, 8, or 13.

Outside of the main cluster cliques, there are five cliques (3, 7, 11, 12, and 15) that are correlated negatively with pH. Cliques 7 and 11 are interconnected and tend to follow similar trends. Cliques 7 and 11 include ASVs associated with the taxonomic classes Acidimicrobiia, Thermoplasmata, Deinococci, and Aquificae. In this case, Deinococci is represented by *Meiothermus*, and Aquificae is represented by *Hydrogenobaculum*. Cliques 7 and 11 are correlated positively with total ammonia and DOC concentrations but are correlated negatively with pH and temperature. The only major differences in trends for cliques 7 and 11 are displayed in Supplementary Figure 6, where clique 7 has a strong negative correlation with nitrate, whereas clique 11 has a slight positive correlation with nitrate.

The unconnected cliques, 3, 12, and 15, are also correlated negatively with pH. Cliques 3, 12, and 15, which contain the taxa *Meiothermus*, Acidimicrobiia, Gammaproteobacteria, Alphaproteobacteria, and Thermoprotei, are correlated positively with total ammonia and DOC concentrations. However, cliques 3 and 12 are correlated negatively with pH but not temperature, whereas clique 15 is negatively correlated with both temperature and pH.

In contrast to the previously mentioned cliques, cliques 17 and 18 are correlated positively with an increase in pH and are outside of the central cluster. Clique 18, which only contains two taxa, Fervidicoccaceae and *Ignisphaera*, a Thermoprotei, is positively correlated with both temperature and pH but negatively correlates with both total ammonia and DOC. Clique 17 contains Nitrososphaeria and Thermoprotei and is correlated positively with temperature, but has no strong correlation with total ammonia or DOC.

The remaining cliques, 1, 6, 10, 14, and 16, lack strong correlations with pH in either direction. Cliques 6, 10, and 16 are only correlated with temperature, cliques 6 and 16 correlated negatively with temperature, and clique 10 correlated positively with temperature. Clique 6 is correlated negatively with temperature and total ammonia concentration but is correlated positively with DOC. Besides temperature, cliques 10 and 16 cannot be differentiated based on pH, total ammonia, or DOC. However, clique 16 is correlated negatively with sulfide and is positively correlated with nitrate (Supplementary Figure 6). There are only 2 nodes in clique 16 representing the phylum Armatimonadota and the genus *Thermoflavifilum*, whereas clique 10 contains the taxa Geoarchaeales and Corynebacterium. Clique 14 is unique in that it is correlated negatively with temperature but has a slight positive correlation with total ammonia. The taxa in clique 14 include *Meiothermus*, Betaproteobacteria, and Gammaproteobacteria. Clique 1, which consists of *Mycobacterium* and *Thiomonas*, is correlated positively with temperature and correlated negatively with total ammonia, but is not strongly correlated with temperature or DOC.

Overall, we find internal consistencies between the pH of samples where ASVs are abundant and the correlation with pH in the co-occurrence networks. For example, taxa associated with higher pH samples in Figure 3 are located within the 6 cliques in the main cluster in Figure 5 and are correlated positively with pH. Of the twelve remaining cliques surrounding the cluster, five of them are negatively correlated with pH, including 3, 7, 11, 12, and 15, and include taxa associated with low pH samples in Figure 3. Cliques 17 and 18 are correlated positively with pH and contain taxa associated with circumneutral to basic samples, as well as Thermoprotei, a taxonomic class found in all hot springs sampled. Finally, the remaining cliques, 6, 10, and 16, show no strong correlation with pH in either direction and contain taxa such as *Meiothermus*, from the class Deinococci, and Armatimonadota, both of which are found across all hot springs sampled.

## 4. Discussion

The stark visual differences in microbial community compositions above versus below the photosynthetic fringe of hot spring outflows were also observed in the NMDS analysis in the distribution of points relative to their location across the photosynthetic fringe, as determined by LDA analysis (see section 2. Material and methods; Figure 4). The difference in the microbial community composition above versus below the photosynthetic fringe was determined and verified to be statistically significant (*p* < 0.05) through an ANOVA test of the CCA axes (Supplementary Figure 5). In contrast, metrics of alpha diversity showed no significant correlation to the LDA-determined photosynthetic fringe (Supplementary Figure 7).

The photosynthetic fringe is not necessarily indicative of an ecotone as described by Meyer-Dombard et al. (2011) (Supplementary Table 1), which is a region where there is either an increase or a decrease in biological diversity where two or more communities mix (van der Maarel, 1990; Meyer-Dombard et al., 2011). Because the photosynthetic fringe represents a transition from a chemotrophic microbial community to a community also consisting of phototrophs, there is the potential that this transition promotes biological diversity. Ecotones have been identified at the photosynthetic fringe of hot spring outflows with streamer biofilm communities, but ecotones were not present at the photosynthetic fringe of hot spring outflows lacking streamer biofilm communities (Meyer-Dombard et al., 2011). Instead, hot spring outflows lacking streamer biofilm communities had higher diversity below the photosynthetic fringe transition. It should be noted that Meyer-Dombard et al. (2011) measured diversity by species richness and taxonomic complexity, whereas the measurement of diversity for this study (Shannon Diversity Index) accounts for both species richness and relative abundances. Overall, Shannon diversity values at the photosynthetic fringe were not higher than values above or below the photosynthetic fringe (Supplementary Figure 7). Instead, the mean Shannon diversity values were lower at the photosynthetic fringe, but not with statistical significance.

Beta diversity was investigated using NMDS analysis with the addition of geochemical vectors. Geochemical factors known to affect microbial community composition, as described in the introduction, including temperature, pH, total sulfide, total ammonia, nitrate, DIC, DOC, and DO concentrations, were added to the NMDS as geochemical vectors (Cox et al., 2011; Hamilton et al., 2011a; Havig et al., 2011; Inskeep et al., 2013). Each of these geochemical parameters, except for total sulfide, exhibited a statistically significant correlation with the overall microbial community composition. Additionally, there were observable patterns among cliques of ASVs and the geochemical parameters determined to correlate with microbial community composition with statistical significance (Figure 5). However, only individual nodes are correlated with sulfide and nitrate concentrations.

Both pH and temperature correlated with microbial community composition with statistical significance according to the NMDS analysis (*p* < 0.05) and were determined to contribute 7.33 and 4.85%, respectively, to variations in the microbial community compositions according to RDA, in agreement with previous studies (Inskeep et al., 2013; Colman et al., 2016; Figure 4 and Supplementary Table 4). The extent of the roles of pH and temperature in affecting microbial community compositions are revealed in the co-occurrence network analysis as well as in the distribution of individual taxa in the 16S rRNA gene amplicon analysis (Figures 3, 5, respectively). In acidic hot spring outflows, classes including Thermoplasmata, Acidimicrobiia, and Gammaproteobacteria are major constituents. In basic hot spring outflows, Cyanobacteria, Chloroflexi, and Deinococci are the major constituents. There are also taxonomic classes present in all samples regardless of pH, such as Aquificae and Nitrososphaeria. These findings are reflected in most of the cliques being correlated with pH (13 cliques) and/or temperature (16 cliques), whereas only 5 cliques are not correlated with pH and only two cliques are not correlated with temperature. There are no cliques that are not correlated with either temperature or pH. Although temperature and pH do contribute to microbial community composition, our analyses indicate that other geochemical factors are additionally contributing to the overall microbial community composition in hot spring outflow communities.

Sulfide concentrations are known to negatively impact oxygenic photosynthesis and have been linked to differences in microbial community composition (Cox et al., 2011; Hamilton et al., 2011b; Boyd et al., 2012; Inskeep et al., 2013; Jørgensen and Nelson, 1988), where the mechanism of sulfide’s negative impact on phototrophs is most likely due to an inhibition of photosystem II (Oren et al., 1979; Miller and Bebout, 2004; Boyd et al., 2012). Rates of autotrophy in Yellowstone microbial mats have also been shown to be sulfide dependent, where acidic phototrophic communities were suppressed at 5 μM sulfide (Boyd et al., 2012). This suppression was not observed in basic mats. The suppression of acidic phototrophic communities at 5 μM sulfide, largely consisting of phototrophic algae, may be due to H_2_S being the dominant form of sulfide in acidic environments. H_2_S has been shown to more readily cross the cell membrane than HS^–^ (Howsley and Pearson, 1979). Our samples were distributed across sulfide concentrations (below detection <0.15 μmolal to 52.6 μmolal) and pH (1.92–9.04). Two samples designated as being at the photosynthetic fringe occurred beyond the previously noted maximum range for photosynthesis of ∼15 μmolal (Cox et al., 2011), thereby expanding the possible sulfide range for photosynthesis in YNP hot springs (Figure 2B). Additionally, eukaryotic phototrophs have been identified in acidic samples with sulfide concentrations above 5 μM, the previously noted limit for phototrophic activity in acidic conditions (Boyd et al., 2012; Romero, 2018). Therefore, the concentrations of sulfide required to limit the presence of phototrophs in YNP hot springs may be higher than initially determined based on measurements coinciding with visual detection of the photosynthetic fringe or measurements of DIC uptake. However, in this study we only observed presence, not activity, under these sulfide concentrations.

When considering the overall microbial community composition, sulfide concentrations did not contribute to the composition with statistical significance even in acidic hot spring outflows (pH < 4, *p* = 0.30) (Figure 4). Additionally, sulfide concentrations only contributed 1.10% to overall variation in microbial community composition according to RDA. The lack of sulfide’s statistical significance in microbial community composition is further supported by the co-occurrence network (Supplementary Figure 6), in which there are no trends between overall clique membership and sulfide concentrations. However, there are individual ASVs, or nodes, within cliques that are correlated with sulfide concentrations, including those representing taxa of known phototrophs, although this does not hold true for all nodes representative of phototrophic taxa. Overall, these findings corroborate the importance of sulfide concentrations in determining the distribution of individual taxa, but do not support the importance of sulfide concentrations in determining overall microbial community compositions, despite previous evidence for the suppression of phototrophic activity in acidic environments.

The gradient of increasing DO concentrations down hot spring outflows contributes 2.68% to changes in the microbial community composition according to RDA (Inskeep et al., 2013). The increase in DO down hot spring outflows is connected to the increased solubility of O_2_ gas as temperature decreases and the increasing extent to which the reduced hydrothermal fluids have equilibrated with the atmosphere. In sulfidic systems, DO is quickly consumed in abiotic oxidation reactions, including the oxidation of reduced sulfur compounds (Inskeep et al., 2013). Examples include both the rapid oxidation of sulfide to polysulfides and the oxidation of sulfide to thiosulfate (the fate for up to 33% of sulfide present in pH 6–8 hot springs in YNP) (Nordstrom et al., 2005). The oxidation of reduced sulfide compounds affects the potential niche availability for sulfur-cyclers, therefore potentially contributing to microbial community composition (Nordstrom et al., 2005, 2009; Inskeep et al., 2013). Though DO in our samples is not correlated significantly with sulfide (−0.201 Pearson correlation), DO does increase with a decrease in temperature and is significantly correlated with temperature (−0.779 Pearson correlation). Therefore, the effects of DO on microbial community composition are difficult to disentangle from those of temperature, as observed by others (Inskeep et al., 2013), but cannot be ruled out in influencing overall microbial community composition.

Nitrogen species correlate with the abundance of subsets (specific cliques) within microbial communities, as well as overall microbial community composition down hot spring outflows. Ammonia and nitrate contribute 3.78 and 2.55% to the variation of overall microbial community (Supplementary Table 4). Furthermore, by including correlations with ammonia and nitrate concentrations in the co-occurrence network analysis, cliques can be further distinguished. Although clique 5 is not correlated with ammonia, it is 1 of only 3 cliques containing nodes representative of known ammonia-oxidizers, the other two cliques being cliques 2 and 13, both of which are correlated negatively with total ammonia. Only two taxa of ammonia-oxidizers, *Nitrosocaldus* and Nitrososphaeria, were identified in the 46 samples. Similar trends are present with nodes representing taxa of known phototrophic nitrogen fixers, such as *Leptococcus*, which trend negatively with ammonia and trend positively with pH. Spring pH could be a driver for N-cycler distribution, but acidic springs provide higher concentrations of N-compounds, such as ammonia, providing what seems to be a sparsely attended buffet for N-cyclers beyond N-fixers. Given these findings, the hypothesis by Hamilton et al. (2014) that ammonia-oxidizers consume the low levels of fixed nitrogen available in basic springs, leaving a niche for nitrogen fixers, would not apply in acidic YNP springs. Additionally, in acidic YNP hot springs, though *nif* genes and N-fixers are found abundantly, ammonia-oxidizers have not yet been identified definitively nor cultured from acidic YNP hot springs (Reigstad et al., 2008; Hamilton et al., 2011a,b; Boyd et al., 2013). However, the *amoA* gene responsible for the first step of ammonia oxidation has been identified in acidic hot springs but is less abundant than in circumneutral to basic hot springs (Boyd et al., 2013). Thus, the cycling of nitrogen in acidic YNP hot springs is ripe for further investigation to characterize the full nitrogen cycle.

According to the NMDS analysis, both DIC and DOC concentrations contribute significantly to differences in microbial community composition along NMDS1 with DIC and DOC contributing 8.20 and 3.60%, respectively, to the variation in the overall composition of microbial community compositions (Figure 4 and Supplementary Table 4). DIC and DOC concentrations are correlated negatively with each other (−0.69 Pearson correlation) and are both strongly correlated with pH (0.79 and −0.77 Pearson correlations, respectively). The strong correlation between DIC and pH is attributed to the increased DIC input in high temperature, circumneutral to basic hot springs, due to being predominantly hydrothermally fed (Nordstrom et al., 2005). Additionally, there is the degassing of CO_2_, which increases the pH for already circumneutral to basic hot springs (Nordstrom et al., 2005). In contrast, the strong negative correlation between DOC and pH has been attributed to increased soil input in acidic springs compared to basic springs which are commonly raised and/or surrounded by sinter, restricting DOC inputs to hydrothermal and microbial sources (Nye et al., 2020).

The effects of dissolved inorganic and organic carbon on microbial community composition are difficult to deconvolve; however, in hot spring outflow studies dissolved carbon and position along the photosynthetic fringe does correlate with changes in the presence or absence of genes associated with specific carbon cycling pathways (Havig et al., 2011). The dominant carbon fixation pathway down the outflow of BP was the reverse tricarboxylic acid cycle, a chemoautotrophic carbon fixation pathway, but below the photosynthetic fringe, the reductive pentose phosphate cycle, a carbon fixation pathway used by oxygenic phototrophs, also became abundant. The transition in the abundance of chemotrophic- versus phototrophic- associated carbon fixation pathway genes is not only indicative of changes in carbon metabolism down hot spring outflows but would be reflected in the microbial community composition. Therefore, at least in the case of a basic outflow, changes in DIC and DOC concentrations are reflected within the microbial communities.

In the present study, the significance of DIC and DOC in microbial distribution is reflected in the clique analysis, with cliques 2–9, 11, and 13 correlating with DOC concentrations (Figure 5). Clique 2 has the strongest negative correlation with DOC and includes taxa found in the outflows of basic sites, where DIC concentrations are comparatively high, and it includes taxa that are generally known as heterotrophs or phototrophs, such as Armatimonadota and *Synechococcus*. In contrast, clique 7 has a strong positive correlation with DOC and includes taxa found typically in cooler acidic sites but are generally known as chemoautotrophs, such as *Hydrogenobaculum* and Acidimicrobia. Even though DIC and DOC concentrations may be important down individual hot spring outflows, when analyzing the NMDS and breaking up the microbial data into cliques for co-occurrence analysis, it becomes more difficult to separate DIC and DOC from pH and temperature to gain meaningful information on the overall composition of hot spring microbial communities.

## 5. Conclusion

Although prior studies have focused on temperature, pH, and sulfide as determining factors for the position of the photosynthetic fringe, these geochemical variables, nor the geochemical variables included in this study, account for the true complexity of hot spring outflows and their hosted microbial communities. Each of the 12 hot spring outflows included in this study is geochemically complex as well as visually and geochemically unique. Though the stark differences visually observed between the predominantly chemotrophic communities above the photosynthetic fringe and the phototroph-containing communities below the photosynthetic fringe of each outflow were supported by CCA to be different in microbial community composition with statistical significance, statistical patterns in the geochemistry across all photosynthetic fringe locations studied are more nuanced. Even when including DIC, DOC, nitrate, ammonia, and DO concentrations, in addition to pH, temperature, and sulfide, the position of the photosynthetic fringe as defined by LDA could not be completely explained across the 12 hot spring outflows included in this study. While temperature and pH correlate with the composition of microbial communities and the position of the photosynthetic fringe down hot spring outflows, other variables, such as DIC, DOC, nitrate, ammonia, and DO concentrations act as supporting players, and are themselves correlated with pH and temperature. Though the co-occurrence analysis provides further differentiation on distribution based on taxa and geochemistry, especially when analyzing through the lens of dissolved carbon or ammonia concentrations, no single variable or set of variables could significantly predict community composition. However, according to both the NMDS analysis and the co-occurrence network analysis, the concentration of sulfide may play less of a role in overall microbial community composition than previously hypothesized. Overall, these findings mirror the complexity of the hot spring outflows studied. Further work inclusive of energy supplies and the rate of change in chemical concentrations down the hot spring outflows were not considered in this study but have been suggested as potential contributing factors in microbial community composition in hot spring outflows (Shock and Holland, 2007; Cox et al., 2011). Furthermore, these are active biological systems where factors of competition and adaptation could also explain departures from what chemical observations might predict (Leibold et al., 2022).

## Data availability statement

The datasets presented in this study can be found in online repositories. The names of the repository/repositories and accession number(s) can be found in the article/Supplementary material.

## Author contributions

ES, GB, and KF conceived the study and supervised the sampling. AH, FD, and GG carried out the DNA extractions, library prep, and sequencing efforts. KW, AH, and ET-R performed the sequence processing and bioinformatic analyses. KF performed the processing of water samples and ion chromatography (IC). KW and ET-R conducted the in-depth statistical analyses of geochemical and microbiomic data, including NMDS, and the co-occurrence network analysis. KW and ET-R wrote the manuscript with input from all authors. All authors contributed to the article and approved the submitted version.

## Footnotes

1
http://www.earthmicrobiome.org/protocols-and-standards/16s/

## Supplementary material

Supplementary Figure 1 Map of Yellowstone National Park depicting the 8 thermal areas (red stars) containing the 12 hot springs sampled as part of this study.

Supplementary Figure 2 A total of 46 samples taken from the outflows of 12 separate hot springs above (blue), at (yellow), and below (green) the photosynthetic fringe displayed across pH and temperature. X marks represent sites in which no Cyanobacteria or Chloroflexia were identified. Circles represent samples in which Cyanobacteria and Chloroflexia were identified, and the size of the circle corresponds to the percent relative abundance (RA) of these bacterial phototrophs in that sample. Asterisks indicate sites in which eukaryotic phototrophs were identified (Romero, 2018).

Supplementary Figure 3 A pairwise matrix analysis of trends between geochemical measurements including temperature, pH, total sulfide, total ammonia, dissolved oxygen, DOC, and DIC. Within each plot in the bottom left of the matrix, each point represents a single hot spring sample. The color of each point indicates whether the sample was taken above (blue), at (yellow), or below (green) the photosynthetic fringe. Corresponding with each plot, a box in the upper right of the matrix lists the overall Pearson correlation between the paired geochemical parameters, as well as the breakdown of the Pearson correlation between the paired geochemical parameters and the relative location of the samples to the photosynthetic fringe as determined by LDA.

Supplementary Figure 4 Percent relative abundance of the 16S rRNA gene sequencing results to the family level. Families not occurring at >20% relative abundance, when summed across all samples, were binned into the “Other” category. Organization of hot spring sites, separated by black bars, follows the order of increasing pH as in Supplementary Table 1. Within each site, samples are organized down the outflow with above (blue diamond), at (yellow diamond), and below (green diamond) the photosynthetic fringe indicated as in Figure 1.

Supplementary Figure 5 Canonical correspondence analysis (CCA) using the 16S rRNA gene sequencing data from each of the 46 sample sites separated by relative position to the photosynthetic fringe. Sample point colors (blue, yellow, and green) refer to the position along the photosynthetic fringe (above, at, below, respectively). Samples above the photosynthetic fringe are separated from samples at or below the photosynthetic fringe on the CCA1 axis with statistical significance (*p* < 0.05) as determined by an ANOVA test. Samples at and below the photosynthetic fringe are separated on the CCA2 axis but not with statistical significance.

Supplementary Figure 6 Co-occurrence network analysis of commonly occurring ASVs within the 46 hot spring samples. The ASVs are organized into 18 cliques using Louvain’s membership algorithm. Edges represent a >0.7 Spearman’s correlation coefficient. Each node is color-coded based on the strength of the Spearman correlation coefficient, negative 1 to positive 1 (blue to red, respectively) between the presence and abundance of the ASV with the following geochemical parameters, **(A)** pH, **(B)** temperature, **(C)** total ammonia, **(D)** DOC, **(E)** total dissolved sulfide, **(F)** nitrate, and **(G)** in relation to the photosynthetic fringe. Panel **(H)** shows all cliques differentiated by color and number of the clique.

Supplementary Table 1 Geochemical data, alpha diversity, and position in respect to the visually determined photosynthetic fringe for each sample. Samples are listed in the order in which they appear in Figure 3.

Supplementary Table 2 Extended geochemical data and position in respect to the visually determined photosynthetic fringe for each sample. All officially recognized hot spring names are listed along with citations. Samples are listed in the order in which they appear in Figure 3.

Supplementary Table 3 Sequence count data for each sample. The raw sequence count, the cleaned sequence count, and the sequence count after normalization are included for each sample.

Supplementary Table 4 Percent contribution for each geochemical variable included in this study on overall microbial community composition determined by RDA. Variables that were determined to contribute to overall community composition with statistical significance (*p* < 0.05) according to the NMDS analysis are bolded. Percent contributions calculated from a subset of sample data are marked with an asterisk.

Supplementary Table 5 ASV count table using the normalized sequence counts for each sample. The SILVA database was used to provide the taxonomic labels for each ASV using QIIME2 (v. 2020.2).

Supplementary Table 6 ASV node table organized by the assigned clique. All nodes present in the co-occurrence network analysis are provided in this table along with the associated ASV down to the lowest available taxonomic classification. The level of taxonomic classification for each ASV is also noted.
