# Supplementary figures and images for: Characterization of microbiomic and geochemical compositions across the photosynthetic fringe
Source: Front Microbiol. 2023 Apr 28;14:1176606. doi: 10.3389/fmicb.2023.1176606 (PMC10178925; doi:10.3389/fmicb.2023.1176606)

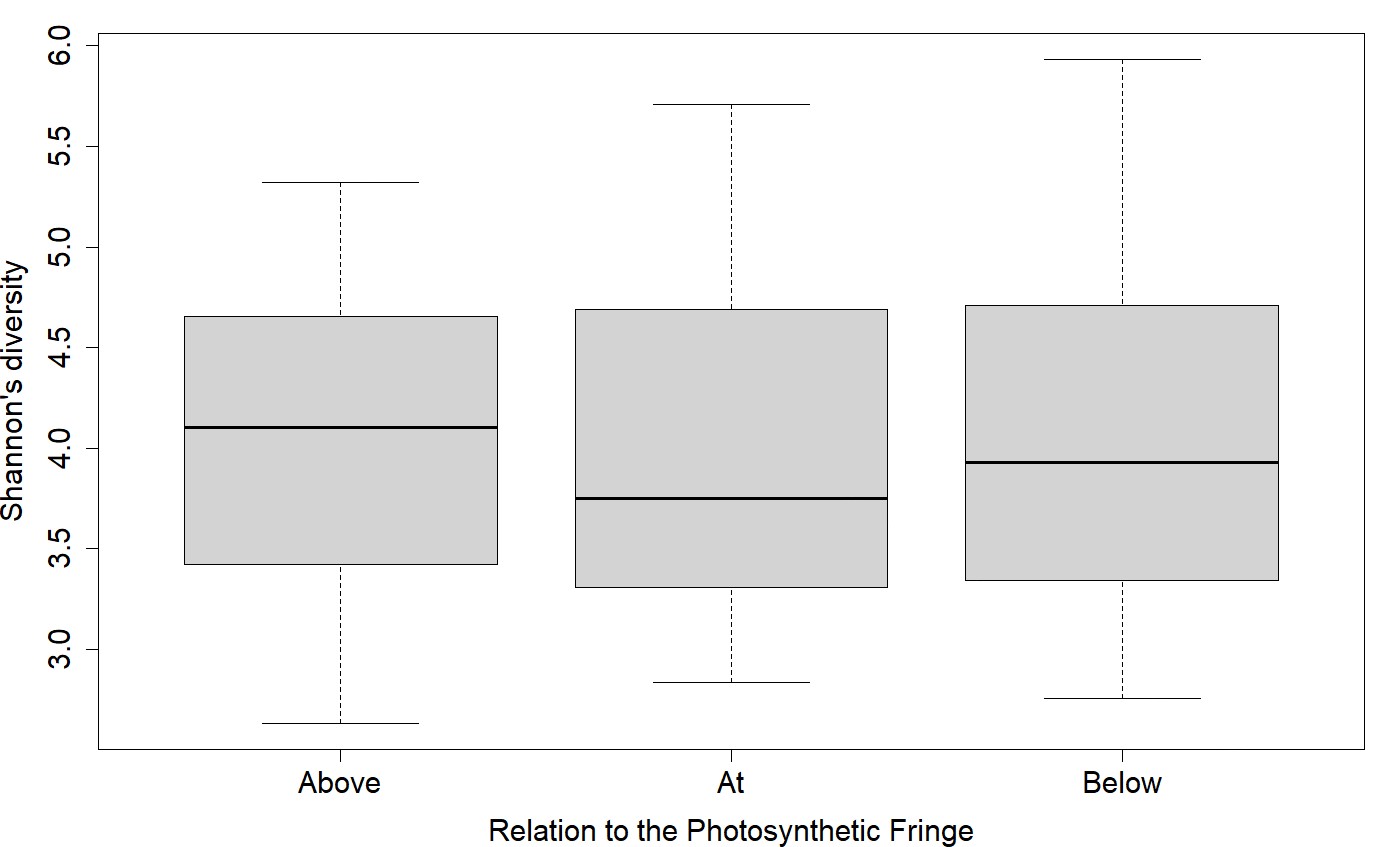

Supplement: Supplementary file 1 [file Image_7.JPEG]

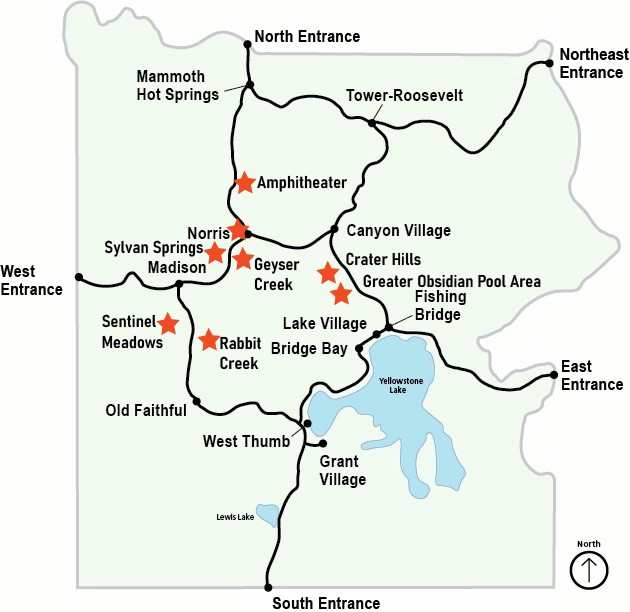

Supplement: Supplementary Figure 1 — Map of Yellowstone National Park depicting the 8 thermal areas (red stars) containing the 12 hot springs sampled as part of this study. [file Image_1.JPEG]

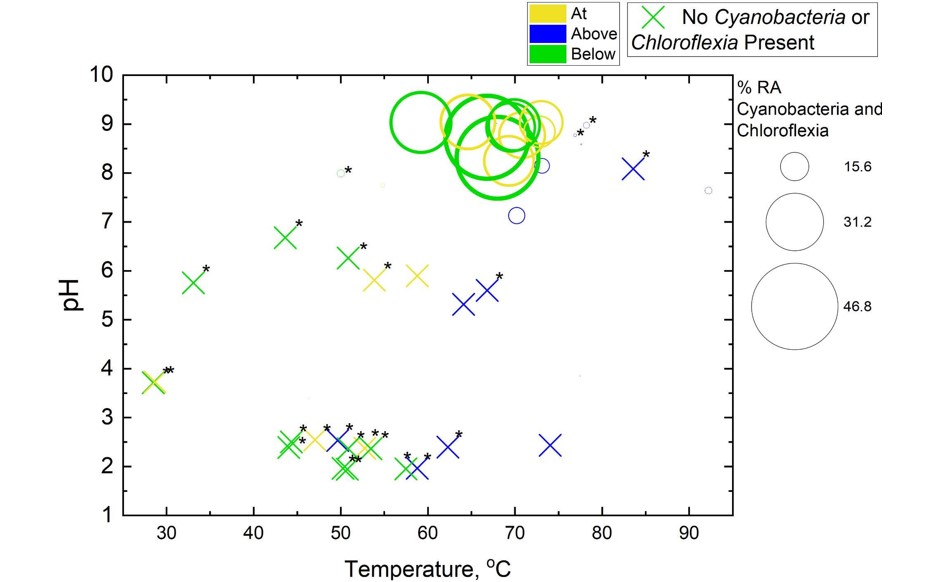

Supplement: Supplementary Figure 2 — A total of 46 samples taken from the outflows of 12 separate hot springs above (blue), at (yellow), and below (green) the photosynthetic fringe displayed across pH and temperature. X marks represent sites in which no Cyanobacteria or Chloroflexia were identified. Circles represent samples in which Cyanobacteria and Chloroflexia were identified, and the size of the circle corresponds to the percent relative abundance (RA) of these bacterial phototrophs in that sample. Asterisks indicate sites in which eukaryotic phototrophs were identified (Romero, 2018). [file Image_2.JPEG]

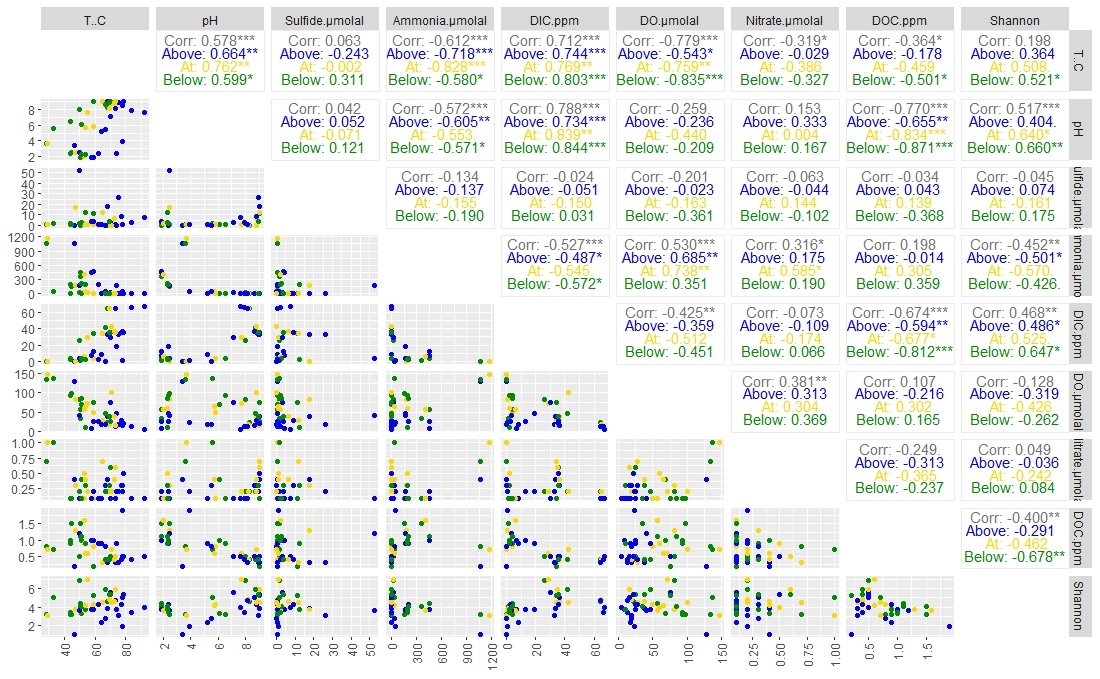

Supplement: Supplementary Figure 3 — A pairwise matrix analysis of trends between geochemical measurements including temperature, pH, total sulfide, total ammonia, dissolved oxygen, DOC, and DIC. Within each plot in the bottom left of the matrix, each point represents a single hot spring sample. The color of each point indicates whether the sample was taken above (blue), at (yellow), or below (green) the photosynthetic fringe. Corresponding with each plot, a box in the upper right of the matrix lists the overall Pearson correlation between the paired geochemical parameters, as well as the breakdown of the Pearson correlation between the paired geochemical parameters and the relative location of the samples to the photosynthetic fringe as determined by LDA. [file Image_3.JPEG]

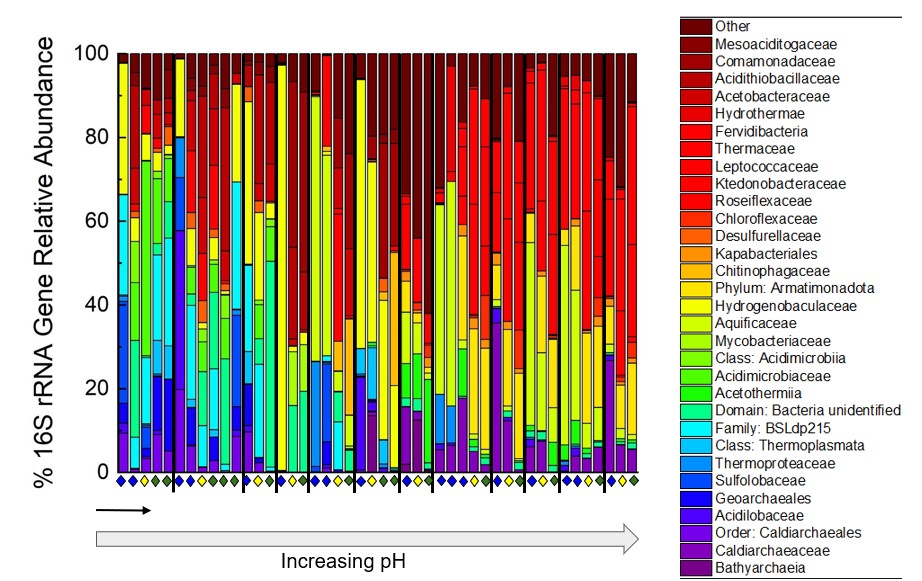

Supplement: Supplementary Figure 4 — Percent relative abundance of the 16S rRNA gene sequencing results to the family level. Families not occurring at >20% relative abundance, when summed across all samples, were binned into the “Other” category. Organization of hot spring sites, separated by black bars, follows the order of increasing pH as in Supplementary Table 1. Within each site, samples are organized down the outflow with above (blue diamond), at (yellow diamond), and below (green diamond) the photosynthetic fringe indicated as in Figure 1. [file Image_4.JPEG]

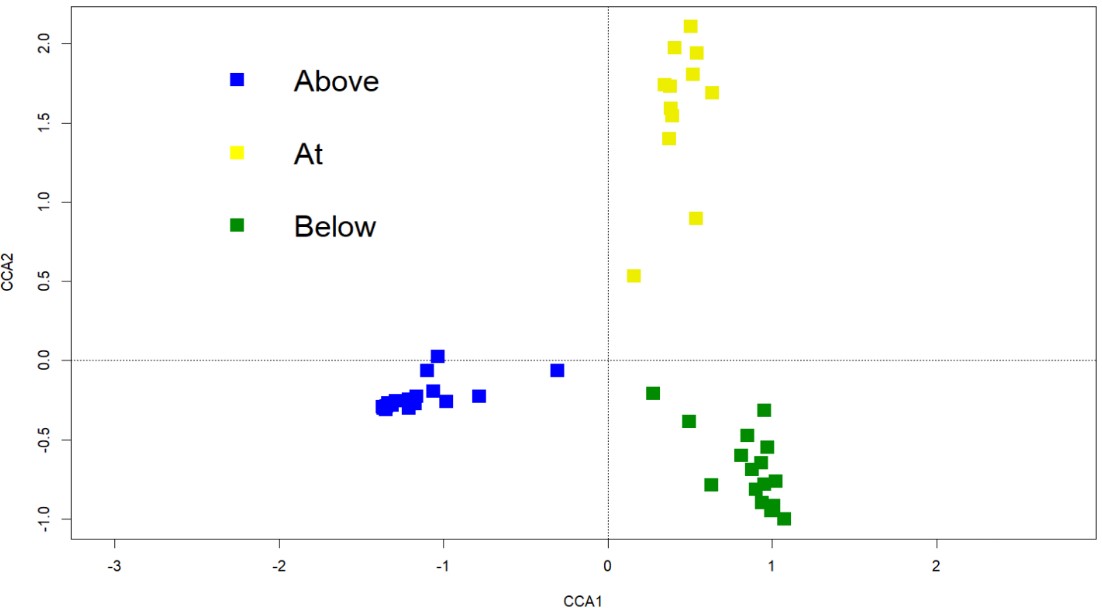

Supplement: Supplementary Figure 5 — Canonical correspondence analysis (CCA) using the 16S rRNA gene sequencing data from each of the 46 sample sites separated by relative position to the photosynthetic fringe. Sample point colors (blue, yellow, and green) refer to the position along the photosynthetic fringe (above, at, below, respectively). Samples above the photosynthetic fringe are separated from samples at or below the photosynthetic fringe on the CCA1 axis with statistical significance (p < 0.05) as determined by an ANOVA test. Samples at and below the photosynthetic fringe are separated on the CCA2 axis but not with statistical significance. [file Image_5.JPEG]

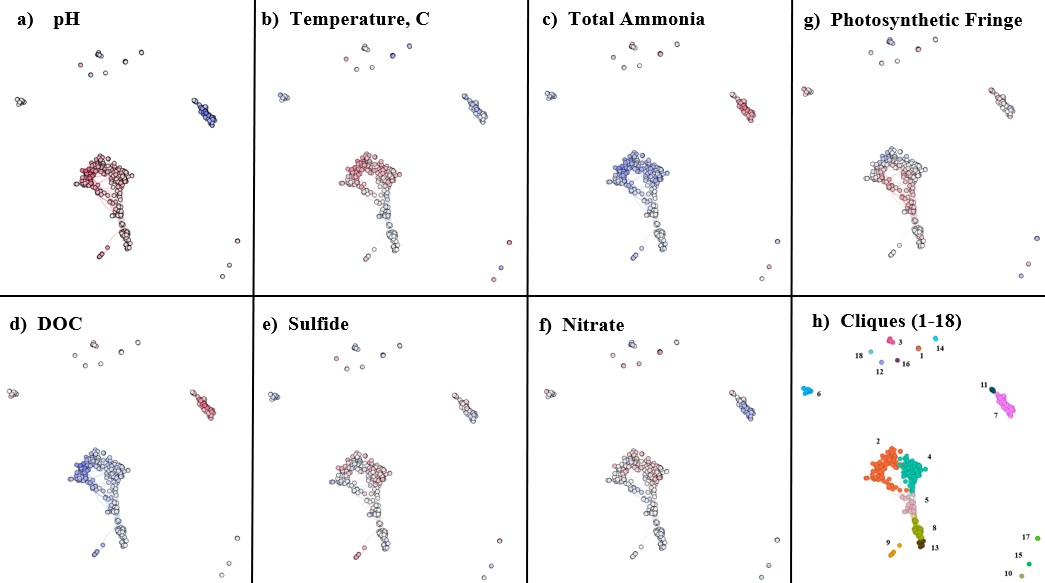

Supplement: Supplementary Figure 6 — Co-occurrence network analysis of commonly occurring ASVs within the 46 hot spring samples. The ASVs are organized into 18 cliques using Louvain’s membership algorithm. Edges represent a >0.7 Spearman’s correlation coefficient. Each node is color-coded based on the strength of the Spearman correlation coefficient, negative 1 to positive 1 (blue to red, respectively) between the presence and abundance of the ASV with the following geochemical parameters, (A) pH, (B) temperature, (C) total ammonia, (D) DOC, (E) total dissolved sulfide, (F) nitrate, and (G) in relation to the photosynthetic fringe. Panel (H) shows all cliques differentiated by color and number of the clique. [file Image_6.JPEG]
